# Assessing national governance of medicine promotion: an exploratory study in Ghana to trial a structured set of indicators

**DOI:** 10.1186/s40545-019-0187-9

**Published:** 2019-09-04

**Authors:** Marcia McLean, Jillian Clare Kohler, Danny Edwards

**Affiliations:** 1 Department of Family and Community Medicine, Dalla Lana School of Public Health, University of Toronto, Toronto, Canada; 2 Leslie Dan Faculty of Pharmacy & Dalla Lana School of Public Health & Munk School of Global Affairs & Public Policy, University of Toronto, Toronto, Canada; WHO Collaborating Centre for Governance, Transparency & Accountability in thePharmaceutical Sector,, Toronto, Canada; 3 Access to Medicine Foundation, Amsterdam, The Netherlands; 4 Utrecht Institute for Pharmaceutical Sciences, Utrecht University, Utrecht, The Netherlands

**Keywords:** Medicine promotion, Pharmaceutical industry, Ghana, Governance, Corruption, Access to medicines

## Abstract

**Background:**

Two billion people worldwide, predominantly in low- and middle-income countries, cannot consistently access required essential medications, thus affecting their ability to attain optimal health outcomes. Access to appropriate medicines may be compromised due to issues involving cost, availability, quality, and prescribing practices, and system-wide factors such as a lack of transparency and accountability. Pharmaceutical promotional practices impact many of these issues, thus influencing the use of appropriate medicines,. Good governance is ultimately the responsibility of national governments through strong health systems with transparent and accountable practices that facilitate appropriate medicine use. We designed a structured set of indicators, based on existing tools, to assess the strength of the national governance of pharmaceutical promotion. In this exploratory study, we trialed the indicators in Ghana.

**Methods:**

Two existing tools, one developed by the World Health Organization and the other by Health Action International with the Medicines Transparency Alliance*,* were adapted to examine the governance of pharmaceutical promotion, resulting in a hybrid framework of 45 indicators of system strength, grouped into four categories: a) Governance of prescription medicines, b) Health care professional codes and regulations, c) Anti-corruption governance, and d) Indexes. Evidence was gathered via desk-based research to establish whether indicator requirements were met.

**Results:**

Our desk-based research discovered the following: a) 21 of 45 indicators for the governance of prescription medicines were met in Ghana, including the existence of a national medicines policy, national medicines list, medicines regulatory authority and a national guide for the promotion of prescription pharmaceuticals; b) pharmacists have a code of conduct specific to ethical promotion though co-development with the pharmaceutical industry should be further examined; and c) anti-corruption indicators were met for 10 of 12 criteria; and d) two indexes were available that were relevant to Ghana.

**Conclusion:**

Our set of indicators identified gaps and opportunities for the governance of medicines promotion in Ghana. These indicators have the potential to highlight areas requiring improved governance and could therefore form a useful diagnostic tool for identifying key discussion points for policy strengthening within low- and middle-income countries.

## Background

An estimated two billion people worldwide, primarily in low- and middle-income countries, 1 cannot access essential medicines due to restricted availability, prohibitive costs, quality issues, or improper prescribing practices [1–3]. Medicines are an essential component of universal health care and have the potential to help health outcomes through improvements in quality of life and reductions in morbidity and mortality [4]. The importance of medicines in achieving global health goals is outlined in Sustainable Development Goal (SDG) #3, *Good Health and Well-Being*, in which target 3.8 specifies that the achievement of universal health coverage includes “financial risk protection, access to safe, effective, quality and affordable essential medicines” [5]. We sought to illuminate how one particular factor affecting appropriate medicine use, medicine promotion, is governed nationally in low- and middle-income countries by creating a set of indicators for rapid diagnosis which we trialed in Ghana.

### Appropriate medicine use and the impact of medicine promotion

Medicine use is considered appropriate when patients receive medicines indicated for their clinical needs at the right dose for the right period of time at the lowest cost [2]. In low- and middle-income countries, over half of medicines are not used appropriately [2, 6]. In addition to the negative impact that inappropriate use has on population health, significant financial consequences result as medicines account for up to 67% of health expenditures in low- and middle-income countries, thus consuming portions of health care budgets that could be otherwise allocated [2, 6].

Ensuring appropriate medicine use involves multiple components of the health system, including registration, financing, selection, procurement, prescribing and dispensing [1, 2]. Quality information that is adequate, correct and unbiased is essential at each phase for rational decisions to be made by government officials, health care providers and patients. One factor affecting information transfer is the promotion of products by pharmaceutical companies, which could affect each of these phases [1]. Medicine promotion may be seen as a means to transmit medicine information, but has been identified as a key factor driving profits for pharmaceutical companies through the influence of the drug utilization patterns [1, 7, 8]. Promotional methods include advertising in medical journals, ghost-written articles, media advertisements, interactions with medical representatives, gift-giving, provision of free samples, sponsored continuing education and distributing company-authored medical information [7, 9, 10]. Inaccurate or misleading information could influence governments to choose, citizens to consume or health care providers to prescribe inappropriate or more expensive medicines, resulting in outcomes that are more expensive, sub-optimal or even dangerous and there is a fine line between influence and corruption [1, 7, 9].

### Governance and oversight

Global pharmaceutical good governance initiatives have been introduced by the World Bank, the World Health Organization (WHO), the Medicines Transparency Alliance (MeTA) and the Global Fund, each with a slightly different focus [11]. World Bank projects have focused on procurement and distribution in the pharmaceutical sector and broader health systems strengthening while the WHO’s Good Governance for Medicines Programme (GGM) was created with an overall goal of improving access to medicines [1, 11]. MeTA projects have focused on improving access to medicines through improved transparency and accountability in the pharmaceutical sector [12]. Finally, the Global Fund concentrated largely on the management of medicine procurement for AIDS, tuberculosis and malaria [11]. While these initiatives have created dialogue and change, their scope is broad. Focusing on one specific issue may result in more expedient change [11].

Medicine promotion is controlled through self-regulation in many countries by means of codes and guidelines [8]. Development of national governance may be influenced by international guidelines that have been created for this purpose [8, 13]. The 1988 WHO publication, *Ethical Criteria for Medicinal Drug Promotion*, remains the global standard [9, 14, 15]. However, the criteria are not legally binding on WHO member states nor do they include suggested processes and sanctions for breaches [14]. These guidelines are outdated as new promotional strategies, such as marketing through social media, have been introduced since their development [13]. The pharmaceutical industry is also provided with global guidance through the International Federation of Pharmaceutical Manufacturers and Associations (IFPMA) Code of Practice, most recently updated in 2019 [16]. While adherence to the code is mandatory for members, membership includes only research-based pharmaceutical companies, thus accounting for a large portion of the pharmaceutical market by value though perhaps not scope [8, 17]. The IFPMA code has been criticized by Health Action International (HAI) for weak standards and enforcement mechanisms [8].

Additionally, organizations with the mandate to provide oversight over the pharmaceutical system have formed, including the Access to Medicine Foundation (ATMF) and Transparency International (TI). The ATMF analyzes company-specific programs and policies related to medicine access in low- and middle-income countries for 20 of the world’s largest research-based pharmaceutical companies. Ethical marketing and anti-corruption are a key theme within the Market Influence and Compliance technical area in the biannually published Access to Medicine Index (ATMI) [18, 19]. TI works to fight multi-sectoral corruption, though has launched an initiative specifically focused on pharmaceuticals and healthcare, recognizing that corruption within this sector is “a matter of life and death” [20]. A 2016 report included marketing within the six activities examined for corrupt practices [21].

However, despite the intentions of these criteria, codes, and mechanisms for oversight, robust country-specific regulatory accountability needs to exist and be enforced for the multitude of components of the pharmaceutical system to optimize the appropriate use of medicines. Due to the complexity of the pharmaceutical system, we chose to focus on one particular component, pharmaceutical promotion; recognizing that it is a multifaceted issue in itself. This collection of indicators was developed for low- and middle-income countries due to the preponderance of access issues, the devastating impact that inappropriate prescribing has on health budgets in these resource-limited environments. We also aspire to provide additional context to the work done by the ATMF by developing a broader understanding of the regulatory environments in which companies examined in the ATMI operate. While companies must continue to be held accountable for their actions, governments need to ensure that governance mechanisms are in place, sufficient, balanced and well-implemented. We determined that there was a need to specifically diagnose national governance of pharmaceutical promotion in a condensed manner to allow for future prioritization of nations for a more detailed inquiry. We utilized existing tools, as will be further described, to design a hybrid tool with the purpose of conducting a rapid assessment to examine the theme of promotion of prescription pharmaceuticals as it relates to appropriate medication use.

## Methods

This structured set of indicators was designed to provide an overview of the governance of pharmaceutical promotion in developing countries. The project was initiated in January 2018, at which time two existing documents were adapted to create a hybrid set of indicators: the *WHO Assessment Instrument for Measuring Transparency in the Public Pharmaceutical Sector* and the preliminary methodology developed by HAI and MeTA, *Medicines Promotion: Assessing the Nature, Extent and Impact of Regulation* [1, 14]. Both documents address issues within the pharmaceutical industry more broadly than the current study intended: the WHO Assessment Instrument is designed to probe transparency and vulnerability to corruption in eight pharmaceutical sector functions from research and development to service delivery, while the HAI/MeTA methodology expands beyond document collection and includes an interview template to guide discussions with key informants.

These tools were combined in this exploratory study to create a hybrid set of indicators to direct the desk-based collection of key publicly available documents focused on promotional practices for prescription pharmaceuticals. Choosing a focused set of indicators from these documents was intended to balance comprehensiveness and pragmatism; sufficiently informative to provide insight while narrow enough in focus to minimize resource requirements. One of the members of our group performed the initial selection of indicators, while the other two provided input on draft versions. From the WHO instrument, indicators were selected from *Section IV: Questionnaire on Medicine Promotion Control* if it was thought possible that the answer could be obtained through a desk-based search. For example, sections IV.1 through IV.10 were included, while sections IV.11 to IV.16 were not included as they contain terminology such as “To what extent do you agree…” and “In your opinion…” The *Document Check List* and *Data Compilation* sections of HAI/MeTA Methodology were reviewed to identify common content and indicators that could contribute to the knowledge of the governance structure surrounding medicine promotion. The indicators chosen were limited to the following broad thematic areas: a) Governance of prescription medicines, b) Health care professional codes and regulations, c) Anti-corruption governance, and d) Indexes.

Ghana was chosen as our starting point as required documents were available in English, thus facilitating analysis, and previous work within the Ghanaian health system indicated the availability of adequate information for assessment. The search for policy documents involved a review of the websites of relevant stakeholders in Ghana, including, but not limited to, the Ministry of Health (MOH), The Food and Drug Authority (FDA), and National Health Insurance Authority (NHIA). Relevant documents from the WHO, MeTA, World Bank and HAI were reviewed. A literature search was conducted using PubMed and Google using the search terms “Ghana,” “drug promotion,” “medicine promotion,” “drug advertising,” “drug promotion,” “pharmaceutical marketing,” “food and drug authority,” “regulation,” “payments health care professionals” in various combinations. There were no limitations placed on time frame.

## Results

### Governance of prescription medicines

Of the 45 indicators under this category, documentation could be found to support 21 (see Table 1). The National Drug Policy (NDP), Second Edition (2004) issued by the Ghana National Drug Programme, a division of the MOH, is intended to guide stakeholders involved in the pharmaceutical sector to ensure that citizens have access to good quality, affordable medicines [22]. Appropriate drug use, one if its key objectives, recognizes the potentially positive impact of health professionals and the negative impact by drug promotion campaigns and dispensing by untrained prescribers [22]. The FDA has published a National Medicines List (NML), a listing of all registered medicines, on its website, though the date of the last update is not stated [22, 23]. An Essential Medicines List (EML), a listing of priority medicines for the population, has been posted on the MOH website, though the most recent version available there is dated 2010 [43]. There appears to be a more recent version (2017) that was accessed via a link found through review of association websites [24].

**Table 1:** Application of the structured set of indicators for assessing governance of the promotion of prescription medicines and anti-corruption in Ghana

Indicator	Ghana
a) Governance of Prescription Medicines	
National Medicines Policy	Yes [22]
National Medicines List	Yes [23]
- Date of most recent update	Not specified
- Does it specify the manufacturer name?	Yes
- Does it specify the country of manufacture?	No
Essential Medicines List	Yes [24]
Medicine regulations include pharmaceutical promotion and advertising	Yes [25]
Medicines Regulatory Authority (MRA)	Yes - FDA
National code/guideline for the ethical promotion of prescription pharmaceuticals	Yes [26]
Does the code explicitly mention?	
- Healthcare professionals	No
- Medical institutions	No
- Patient organizations	No
- Medical representatives	No
- The general public	Yes
- Media	Yes
Does it cover the following forms of promotion?	
- Direct-to-consumer advertising	Yes
- Disease awareness campaigns	No
- Interactions with medical representatives	No
- Speakers fees and consultancies	No
- Continuing education sponsorship	No
- Prescription samples	No
- Patient information	No
- Packaging, labeling and package inserts	No
- Promotion of off-label indications	Yes
- Gift-giving	Yes
Is pre-approval required?	Yes
Does the pre-approval submission require the following information:	[27]
- International non-proprietary name (INN)	Yes
- Brand name	Yes
- Company name	Yes
- Major indications for use	No
- Adverse effects	Yes
- Contraindications	No
- Medicine interactions	No
- Cost	No
Is monitoring in force?	Yes
Is there a formal mechanism for lodging a complaint?	No
Are enforcement and sanctions stated?	No
Are violations publicly available?	Yes
Is there a government-sponsored, unbiased source of drug information?	No
National Pharmaceutical Industry Association	Yes [28]
- Represent multi-national manufacturers	No
- Represent national manufacturers	Yes
- Associated with IFPMA	No
- Code of conducts specific to ethical promotion	No
b) Health Care Professional Codes and Regulations	
National Pharmacy College Code of Conduct specific to ethical promotion	Yes [29]
National Physician College Code of Conduct	No [30]
c) Anti-Corruption Governance	
Signing of the UN Convention Against Corruption (UNCAC)	Yes [31]
Signing of UN Convention on Human Rights	17/18 treaties [32]
National Anti-Corruption Law(s)	Yes [33]
African Union Convention on Preventing and Combatting Corruption	Yes [34]
Economic Community of West African States Protocol on Corruption	Yes [35]
National Anti-Corruption Strategy	Yes [36]
National Anti-Corruption Commission	Yes [37]
National Law on Freedom of Information	No
Whistleblower Protection	Yes [38]
Transparency Laws	No
Code of Conduct for Civil Servants	Yes [39]
Ethical Principles for Civil Servants	For GHS [40]
d) Indexes	
Corruption Perception Index (Transparency International)	40; 81/180 countries [41]
Regional and/or National Indexes (if available)	IIAG 65; 8/54 [42]

*The Public Health Act (2012; Act 851)* confers responsibility for drug regulation to the FDA, including regulating the promotion of prescription medicines [25]. The Act specifies that labeling, packaging and advertisements may not be misleading to consumers with respect to its value or merits and that promotional materials and advertisements must be pre-approved by the FDA. The FDA has published guidelines [26] outlining the details for pre-approval submissions and prohibited forms of advertising. The regulations state that advertisements should cautiously advise drug use with enough information to allow a risk-benefit assessment and appropriate medicine use should be stressed [26]. The regulations do not mention interactions with medical representatives, medical samples, continuing education activities, patient information, packaging and labeling nor gift giving.

The pre-approval application form posted by the FDA specifies the criteria for the advertisement including type of product, dosage form and brand and generic names [27]. However, the application does not stipulate a requirement for the major indication for use and it is unclear whether mention of adverse effects is required in the advertisement itself. As reported in the FDA 2016 Annual Report, the FDA received 217 drug advertisements, of which it approved 169 and deferred 48 due to “incomplete script” [44]. The FDA has engaged a department for monitoring advertisements, which found 35 unauthorized advertisements resulting in the issuance of 15 cautions, two sanctions and 18 cases reported to police [44]. Though the FDA website contains a comprehensive listing of operational guidelines, it does not detail the process for monitoring advertisements; whether this is an active or passive process [45]. Sanctions are addressed in Section 129 of the Food and Drug Act, whereby contraventions are stated to be subject to fines ranging from 7500 to 15,000 penalty units and/or imprisonment of 15–25 years [25].

### Healthcare professional codes and regulations

Professional codes were found for the guidance of pharmacists but not for physicians. *The Health Professions Regulatory Bodies Act, 2013 (ACT 857)* consolidated legislation for physicians, dentists, nurses and midwives, pharmacy personnel, and allied health professionals and recognized respective regulatory bodies [46, 47]. The Act cannot be found in its entirety in one location: the MOH website does not list the act under its policies; a Google search uncovered a link on the ministry site containing a list of documents, including Part 3 of the Act [47]; and the Pharmacy Council website includes only the portion relevant to pharmacy [48].

Pharmacy personnel involved in medicine dispensing, including pharmacists, pharmacy technicians and over the counter medicine sellers (OTCMS), are licensed by the Pharmacy Council. The Council is also tasked with ensuring competency of pharmaceutical care providers and inspecting pharmacies and other establishments for drug dispensing [48–51]. Ethical practice appears has been assumed by the Pharmaceutical Society of Ghana (PSG), a partner organization that includes the Association of Representatives of Ethical Pharmaceutical Industries (AREPI) as one if its five practice groups [29]. Article 9 of the code of ethics for the PSG, contained within the Society’s Constitution, states, “To consider something from AREPI’s code of ethics against unethical and unsolicited promotion” [29]. AREPI does not have a website and the code of conduct could not be found.

### National anti-corruption governance

Strategies to curb corruption in recent decades have included adoption of international, regional and sub-regional initiatives; respectively, the United Nations Convention Against Corruption (UNCAC) in 2003, the African Union Convention on Preventing and Combating Corruption in 2005, and the Economic Community of West African States (ECOWAS) Protocol on the Fight Against Corruption in 2003 [31, 34, 35]. Adoption of these conventions may signify an intention to comply with recognized principles.

The Ghanaian Constitution mandated the Commission on Human Rights and Administrative Justice (CHRAJ) to manage anti-corruption activities [33, 37]. In 2012, the CHRAJ coordinated the development of the National Anti-Corruption Action Plan (NACAP), intended to guide anti-corruption activities in the public and private sectors [36]. Using the words of Kofi Annan, the NACAP describes corruption as an “insidious plague” that corrodes societies, violates human rights and hinders economic growth. The NACAP notes that though efforts have been made in Ghana to strengthen the legal framework to combat corruption, fighting this complex problem requires shifting societal principles so the values of “integrity, transparency and accountability” become mainstream values across sectors [36]. The NACAP notes that “corruption remains endemic in Ghana despite the wide array of measures pursued in the past to control the problem,” including significant corruption in the health care sector, such as inappropriate prescribing [36].

### Indexes

Two indexes can illuminate the quality of governance in general in Ghana: The Corruption Perceptions Index (CPI), issued annually by Transparency International, and the Ibrahim Index of African Governance (IIAG) by the Mo Ibrahim Foundation [41, 42]. In the 2017 CPI, Ghana is ranked 81st out of 180 countries with a score of 40 (from 0 being highly corrupt to 100 being very clean), its lowest score over the past six years [41]. Measurement of government performance according to the IIAG ranks Ghana amongst the top ten African nations (score: 65/100; ranking 8/54); however, it also ranks amongst the top 10 deteriorated countries over the past decade [42].

## Discussion

Ghana has an estimated population of 28 million and is classified as a lower-middle-income country (LMIC); upgraded from a low-income country (LIC) in 2011 [52, 53]. In 2015, approximately 75% of health expenditures were funded nationally and 25% through external sources such as development funds, a portion expected to diminish since the LMIC classification [54]. By 2030, the Ghanaian population is projected to increase and the proportion of people older than 64 is projected to increase significantly, thus shifting the disease profile to chronic disease management and increasing the demand for medicines [55, 56]. Health status disparities exist due to the inconsistent application of universal health care principles across different geographies and income levels, and inefficiencies have been noted with medicine use [56, 57]. Appropriate use of medicines will be increasingly essential to achieve population health goals and to ensure that resources are responsibly allocated. As work on the health system progresses, policy and oversight procedures will need to be developed to ensure a quality, well-functioning pharmaceutical system.

Inadequate pharmaceutical system governance, that is to say, suboptimal transparency and weak accountability mechanisms, have been identified as factors impeding global access to medicine [1]. Governance includes the process of decision-making and implementation and the formal and informal actors involved, including government and civil society [58]. Key components of good governance are transparency, the degree to which information is available and understandable to the public, and accountability, the extent to which agencies are answerable to those affected by their decisions; concepts which are co-dependent [12, 57, 59]. The tool developed in this exploratory study identifies specific indicators for the national governance of pharmaceutical promotion and whether they are publicly available, a proxy for transparency. Though often assumed that corruption results when governance is lacking, the absence of governance in particular areas does not mean that a system is corrupt; rather it highlights areas where corruption could be at greater risk of occurring [1, 60].

We chose to focus on one particular component of appropriate medicine use: the national governance underlying pharmaceutical promotion. Indicators were selected from two existing tools, the *WHO Assessment Instrument for Measuring Transparency in the Public Pharmaceutical Sector* and the HAI/MeTA tool, *Medicines Promotion: Assessing the Nature, Extent and Impact of Regulation*, to create a desk-top research framework that would direct the compilation of publicly available documents [1, 14]. This hybrid framework is intended to impart an understanding of whether countries have taken steps to adapt principles, policies and implement laws in areas identified as important in the area of pharmaceutical promotion.

Our exploration of the theme of pharmaceutical promotion and anti-corruption in the context of Ghana illuminates strengths and weaknesses in governance. While, Ghana does have a National Drugs Policy, the most recent version posted on the MOH website is 2004 [22]. Work on this version appears to be in progress, however it seems that five years have lapsed since the process was initiated [28]. Access to the NML and EML is not straightforward, as outlined above, highlighting a potential issue with transparency and raising the question of utilization of these documents in practice [22–24, 43, 61]. Ghana has legislation to control pharmaceutical promotion, an agency that pre-approves and monitors advertisements, a website that lists breaches and an annual report that indicates that prosecution is underway for some cases [25–27, 44]. However, some forms of pharmaceutical promotion, such as interactions with medical representatives, continuing education sponsorship, patient information and prescription samples are not included in the regulations from the FDA and it is particularly concerning that the national industry association, The Pharmaceutical Manufacturers’ Association of Ghana (PMAG), considers that advising patients on medications is one of their core responsibilities [62]. The Pharmacy Council governs the licensing of pharmacists, pharmacy technicians, licensed medicine sellers and their establishments, however the ethical code of conduct is under the governance of the Pharmaceutical Society of Ghana, their partner advocacy organization and refers its ethical guidance to the code of the association of pharmaceutical manufacturers. The national college of physicians does not have a publicly available code of conduct [30]. Ghana has taken steps to implement anti-corruption and whistleblower legislation and implemented an anti-corruption strategy in 2012 [33, 36, 38]. The National Anti-Corruption Action Plan recognizes corruption as one of the barriers to Ghana’s socioeconomic and political development over past decades [36]. Ghana has put some policies and procedures in place, though areas of governance that could be strengthened were identified. To summarize, while it seems that important efforts have been made to govern pharmaceutical promotion, further examination is required to understand implementation; in other words, is policy put into practice sufficiently well?

This exploratory study suggests that the structured set of indicators developed here could be useful in identifying areas of weak governance, thus providing a starting point for further exploration with key informants and a basis for discussion with governments. By focusing on the specific area of pharmaceutical promotion, research could be focused and the desk-based research method proposed here, would be less resource-intense than the other methodologies examined. Limiting research resources per country could allow for a more expansive exploration, thus providing a broader worldview of the national governance in which multinational pharmaceutical companies operate, and the extent to which the actions of national pharmaceutical companies are monitored.

A key limitation of this study is the lack of validation of document collection by key informants within the Ghanaian health system. We also acknowledge that documents may exist that were not found through the search strategy utilized. It is also not known whether knowledge of each regulation is known by relevant actors within the system nor whether their principles are adhered to in practice. Collaboration with key informants is an essential next stop to more thoroughly understand the pharmaceutical governance structure and the implementation and practical application of policies and legislation. Results are intended to highlight areas of weak governance, ultimately leading to prioritization of future study foci, such key informant interviews and direct engagement with national governments according to gaps identified.

## Conclusions

This exploratory study, utilizing a structured set of indicators for examining national governance of pharmaceutical promotion, showed that directed desk-based research can provide a base from which further discussion with relevant stakeholders could be initiated. We identified some areas within the Ghanaian system where governance could be strengthened and transparency could be improved to ensure that the pharmaceutical system is accountable to the population of Ghana. This rapid assessment method could provide a platform from which to initiate discussions with national governments on strategies to minimize the extent to which pharmaceutical promotion influences appropriate medicine use, thus mitigating the impact that this practice has on the achievement of optimal health outcomes.

## Data Availability

Data sharing not applicable to this article as no datasets were generated or analyzed during the current study.

## Abbreviations

AREPI: Association of Representatives of Ethical Pharmaceutical Industries
ATMF: Access to Medicine Foundation
ATMI: Access to Medicine Index
CHRAJ: Commission on Human Rights and Administrative Justice
CPI: Corruption Perceptions Index
ECOWAS: Economic Community of West African States
EML: Essential Medicines List
FDA: Food and Drug Authority
GGM: Good Governance for Medicines Programme (WHO)
HAI: Health Action International
IFPMA: International Federation of Pharmaceutical Manufacturers and Associations
IIAG: Ibrahim Index of African Governance
INN: International non-proprietary name
LIC: Low-income country (World Bank income classification)
LMIC: Lower-middle-income country (World Bank income classification)
MeTa: Medicines Transparency Alliance
MOH: Ministry of Health
MRA: Medicines Regulatory Authority
NACAP: National Anti-Corruption Action Plan
NDP: National Drug Policy
NML: National Medicines List
OTCMS: Over the counter medicine sellers
PMAG: Pharmaceutical Manufacturers’ Association of Ghana
PSG: Pharmaceutical Society of Ghana
SDG: Sustainable Development Goal
UNCAC: United Nations Convention Against Corruption
WHO: World Health Organization

## Footnotes

1 Countries are classified according to 2017 World Bank data. Low-income countries have a gross national income (GNI) per capita of < $1005 and lower-middle-income countries between $1006 and $3955 GNI/capita (https://blogs.worldbank.org/opendata/new-country-classifications-income-level-2017-2018)
